# Percutaneous mitral valve repair: the necessity to redefine secondary mitral regurgitation

**DOI:** 10.1007/s12471-020-01412-2

**Published:** 2020-03-23

**Authors:** J. Halim, B. Van den Branden, P. Coussement, E. Kedhi, J. Van der Heyden

**Affiliations:** 1 Department of Cardiology, Sint-Jan Hospital, Bruges, Belgium; 2 Department of Cardiology, Amphia Hospital, Breda, The Netherlands

**Keywords:** Mitral regurgitation, Percutaneous mitral valve repair, Heart failure

## Abstract

Interest in percutaneous mitral valve repair has increased during recent years. This is mainly driven by the significant number of patients being declined for mitral valve surgery because of a high risk of surgery-related complications or death. In this subset of patients, percutaneous edge-to-edge repair using the MitraClip device (Abbott, Menlo Park, CA, USA) has become an established treatment option, proven to be safe, efficient and associated with improved functional status. In contrast to primary mitral regurgitation (MR), clinical outcomes after mitral valve surgery appear to be less favourable as regards secondary MR due to heart failure. In the MITRA-FR and COAPT trials, patients with moderate to severe and severe secondary MR with reduced left ventricular function received either medical treatment (control group) or MitraClip implantation plus medical treatment (device group). Results were conflicting, with only the COAPT trial showing better clinical outcomes in the device group. However, both trials are now seen as complementary and provide useful information especially regarding patient selection for MitraClip therapy. The goal of this review is to delineate which subset of patients with secondary MR will potentially benefit from percutaneous mitral valve repair.

## Introduction

Mitral regurgitation (MR) is the second most frequent indication for valve surgery [1]. Worldwide, up to 50,000 operations are performed for MR on an annual basis, with more than half of these including isolated mitral valve surgery (MVS) [2]. Traditionally, MR can be divided into primary and secondary MR. Primary MR is the result of an abnormality in the mitral valve (MV) apparatus. Most commonly, there is degenerative MV disease present with prolapse or flail leaflets. MV prolapse is prevalent in 1–2.5% of the population [3]. On the other hand, secondary MR is caused by disease of the left ventricle (ischaemic or non-ischaemic cause) leading to left ventricular (LV) dilatation and displacement of the papillary muscles. These geometric changes lead to insufficient coaptation of the valve leaflets, resulting in MR [4].

MVS in primary MR has been extensively studied. As we know, in this group of patients the indication for surgery has been clearly defined with better clinical outcome compared to medical treatment alone [5, 6]. However, this does not apply for secondary MR. MVS in these patients is associated with high operative mortality, high recurrence of significant MR and, most importantly, it has not led to a better survival [7–10]. Consequently, it is still not clear whether correction of a secondary MR in patients with heart failure will lead to a better prognosis. MVS in this subset of patients is therefore mostly performed when coronary artery bypass grafting is considered.

Importantly, about 50% of the patients with an indication for MVS are considered not suitable for surgery. These patients are denied surgery due to their age, poor LV function, frailty and significant co-morbidity [11]. In this subset of patients, MV repair has emerged as an eligible treatment alternative. The most widely adopted technique is the edge-to-edge MV repair (MitraClip; Abbott, Menlo Park, CA, USA). The procedure has proven to be safe and is associated with improvement in New York Heart Association (NYHA) functional class and quality of life [12, 13].

More than 70,000 implants have been performed to date worldwide and a large amount of outcome data is available. However, these data are mainly from observational studies whose value is too limited to support clinical indication as in guidelines.

Recently, the findings of two eagerly awaited randomised controlled trials (RCTs) have been published. Both MITRA-FR and COAPT investigated the role of MitraClip treatment in patients with reduced LV function and ischaemic or non-ischaemic secondary MR, who remained symptomatic (NYHA class $\geq$2) despite optimal medical treatment [14, 15].

While the results of these two trials have created more controversy regarding percutaneous MV repair, it has also given us the opportunity to elucidate why such well-designed trials have provided such different results.

## Echocardiography: patient eligibility criteria

**Fig. 1:**
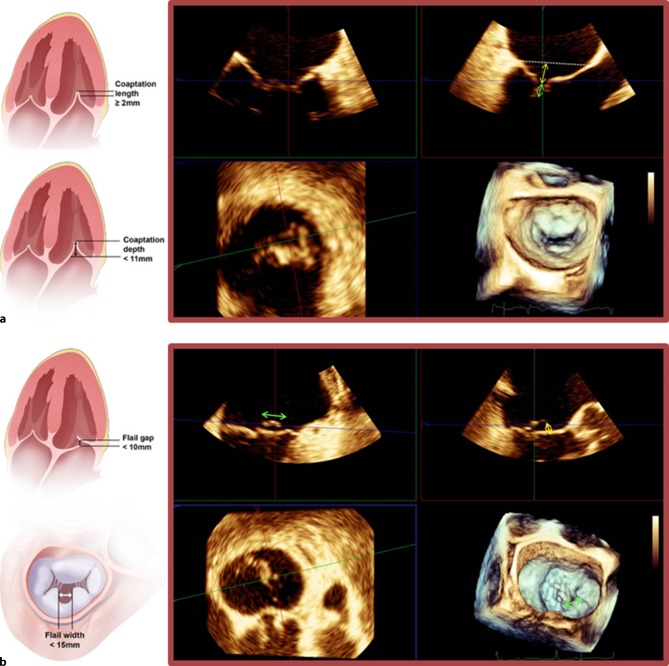
**a**–**c** Transoesophageal echocardiogram: assessment of mitral valve eligibility for MitraClip implantation

**Fig. 2:**
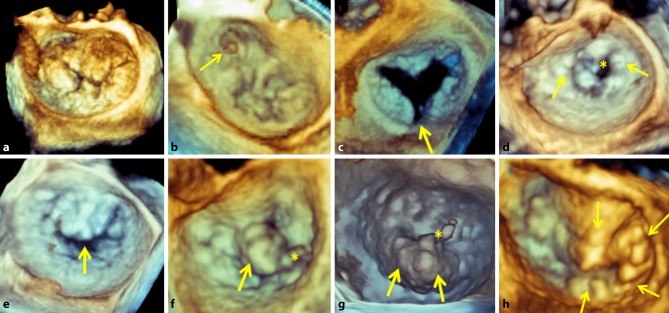
**a**–**h** Additional value of 3D mitral valve echocardiography for anatomical and morphological assessment in screening for MitraClip therapy. All patients presented with severe mitral regurgitation but are anatomically not eligible for MitraClip therapy. **a** Complex Barlow degeneration involving prolapse of all mitral segments. **b** Anterolateral commissural prolapse *(arrow)*. **c** Posterior leaflet cleft *(arrow)*. **d** Severe rheumatic stenosis (*asterisk*) with diffuse calcifications and commissural fusion (*arrows*). **e** Loss of central coaptation during systole *(arrow)*. **f** A2 flail *(arrow) *with chordal rupture (*asterisk*), flail width 17 mm. **g** P2 flail *(arrows) *with chordal rupture (*asterisk*), flail width 22 mm. **h** Complex Barlow degeneration with prolapse of A2, A3, posteromedial commissure and P3 scallops *(arrows)*

Echocardiography still remains the gold standard in evaluating the severity and mechanism of MR. After confirming moderate to severe MR or severe MR the next step is to evaluate if a patient is suitable for percutaneous MV repair. The EVEREST 2 study [12] has clearly defined which specific echocardiographic measurements are associated with a greater reduction of MR (Tab. 1, Figs. 1 and 2).

**Table 1:** Echocardiographic eligibility criteria for MitraClip implantation

	*Suitable morphology*
	Degenerative or functional aetiology
	Mitral valve orifice area >4 cm^2^
	A2-P2 pathology
	Central jet
	Length of posterior leaflet ≥10 mm
	Lack of calcification in the grasping area
Functional MR	Coaptation length ≥2 mm
	Coaptation depth <11 mm
Degenerative MR with prolapse	Flail gap <10 mm
	Flail width <15 mm
	*Unsuitable morphology*
	Significant mitral stenosis
	Multiple and eccentric jets
	Severe calcification in the grasping area
	Rheumatic, endocarditic mitral valve
	Lack of primary and secondary chordal support, perforated mitral leaflets or clefts
	Gap between leaflets >2 mm

*MR* mitral regurgitation

## MitraClip: the EVEREST studies

The EVEREST 1 study, a multicentre, single-arm prospective study, was the first study confirming the safety and efficacy of the MitraClip system. More than half of the patients showed a reduction in MR grade at discharge. Durability was, however, a concern with 30% of the patients requiring MVS within 3 years because of significant MR (grade of ≥3) [12].

The EVEREST 2 study was the first RCT in which patients underwent either MVS (repair/replacement) or percutaneous MV repair (MitraClip system). Importantly, high-risk patients for cardiac surgery were excluded from this study and only 27% of these patients had secondary MR. At 1‑year follow-up, MR grade ≥3 was more frequent in the device group (17.9% vs 0%, *p* = 0.004). Because experience of operators in this study was limited, a second clip was underused. An improvement in quality of life, NYHA functional class and LV dimensions could nevertheless be seen at 1 year in the MitraClip group with no significant difference between the two groups. Moreover, superior safety was seen in the MitraClip group compared to patients undergoing surgery [13]. At 5‑year follow-up, the mortality rate was not significantly different between these two groups. The composite endpoint including survival, MVS or MR grade 3+/4+ was higher in the surgery group than in the MitraClip group (64.3 vs 44.2, *p* = 0.01). The difference was caused by a higher rate of MR grade 3+/4+ and more MVS being performed in the first 6 months after MitraClip implantation. Beyond 6 months there was no difference between these two endpoints. These results confirmed the durability of the MitraClip system [16].

Additional observational studies showed that implantation of the MitraClip device in high-risk patients was safe and effective in reducing the MR grade and in improving the NYHA functional class [17–19]. In patients with secondary MR treated with MitraClip implantation the same results were obtained [20–22].

## MITRA-FR vs COAPT trial

The MITRA-FR and COAPT trials are both multicentre RCTs, in which patients with heart failure (NYHA class ≥2 despite heart failure medication) and moderate to severe MR or severe secondary MR receive either medical treatment (control group) or undergo MitraClip implantation plus medical treatment (device group) [14, 15].

In the MITRA-FR trial, patients had severe secondary MR defined by a regurgitant volume (RV) ≥30 ml/beat or an effective regurgitant orifice area (EROA) of ≥20 mm^2^ assessed by echocardiogram. Furthermore, these patients had a left ventricular ejection fraction (LVEF) between 15% and 40% and symptoms of heart failure (NYHA class $\geq$2). All patients had to be assessed by the heart team and had to be found unsuitable for MVS. In total, 304 patients were included: 152 patients were allocated to the device group and 152 patients to the medical group. All patients were treated with heart failure medication according to the ESC guidelines for patients with reduced LVEF. The composite primary endpoint of death from any cause and unplanned hospitalisation for heart failure at 12 months did not show any significant difference between the two groups (54.6% in the device group vs 51.3% in the medical group; *p* = 0.53) (Tab. 2). Importantly, MR grade was 0/1+ at discharge in 74.6% of the patients, confirming procedural success in most of the patients. Unfortunately, a significant amount of data was missing at 1‑year follow-up. This included echocardiographic outcomes, quality of life outcomes and NYHA functional class.

**Table 2:** Study endpoints of the MITRA-FR and COAPT trial

	Device group	Control group	*p*-value
**MITRA-FR**			
*Primary endpoint*			
Composite outcome of death from any cause or unplanned hospitalisation for heart failure at 12 months (%)	54.6	51.3	0.53
*Secondary endpoint*			
Death from any cause (%)	24.3	22.4	
Cardiovascular death (%)	21.7	20.4	
Unplanned hospitalisation for heart failure (%)	48.7	47.4	
Major adverse cardiovascular events (%)	56.6	51.3	
**COAPT**			
*Primary endpoint*			
All hospitalisations for heart failure within 24 months (%)	35.8	67.9	<0.001
Freedom from device-related complications at 12 months (%)	96.6		<0.001 for comparison with goal of 88%
*Secondary endpoint*			
MR grade 2+ or lower at 12 months (%)	94.8	46.9	<0.001
NYHA functional class 1 or 2 at 12 months (%)	72.2	49.6	<0.001
Change in LVEDV from baseline (ml)	−3.7 ± 5.1	17.1 ± 5.1	0.004
Death from any cause within 24 months (%)	29.1	46.1	<0.001

No *p*-values are reported for secondary endpoints in the MITRA-FR trial due to the fact that no adjustments are made for multiple testing

*MR* mitral regurgitation, *NYHA* New York Heart Association, *LVEDV* left ventricular end-diastolic volume

In the COAPT trial, patients with moderate to severe (grade 3+) or severe (grade 4+) secondary MR were included. Moderate to severe MR was defined by an EROA of ≥30 mm^2^ and/or RV of ≥45 ml. Severe MR was defined by an EROA of ≥40 mm^2^ and/or a RV ≥60 ml.

All of these patients had a LV function between 20% and 50% and heart failure symptoms (NYHA class $\geq$2) despite optimal heart failure medication and resynchronisation therapy if indicated. Patients were deemed unsuitable for surgery on the basis of discussion by the heart team. As an extra check, an eligibility committee confirmed which the patients fulfilled the inclusion criteria (taking maximum doses of guideline-directed heart failure medication) and agreed which patients were not suitable for MVS (STS score of 8% or a high risk of operative stroke or death). A total of 302 patients were included in the device group and 312 patients in the control group. Primary endpoint of this study was all hospitalisations for heart failure within 24 months. This was significantly lower in the device group than in the control group (35.8% vs 67.9%, *p* ≤ 0.001). All-cause mortality was also significantly lower in the device group (29.1% vs 46.1%, *p* ≤ 0.001). Additional secondary endpoints were also in favour of the device group: quality of life, functional capacity, MR grade and LV remodelling. These results could be reproduced in all subgroups, including patients who had ischaemic or non-ischaemic cardiomyopathy. MR severity, LV function and volume also did not influence the outcomes. Lastly, 96.6% of the patients were free of device-related complications, confirming the safety of the MitraClip system.

## MITRA-FR and COAPT trial: who to believe?

With these two RCTs showing such contradictory results the debate has continued as to whether percutaneous MV repair of severe secondary MR in heart failure patients is beneficial.

Since both studies were well executed and show remarkable similarities in design, it is hard to decide who to believe. So why were the clinical outcomes in the COAPT trial in favour of the device group? And why could these results not be reproduced in the MITRA-FR trial? In order to understand these conflicting results, we need to become aware of the subluminal differences first (Tab. 3).

**Table 3:** Important differences between MITRA-FR and COAPT trials

	MITRA-FR	COAPT
**Baseline characteristics and inclusion criteria**		
No. of patients	304	614
Medical therapy at baseline	Variable doses of HF medication	Stable maximum doses of HF medication Resynchronisation therapy if indicated
LVEF (%)	≥15 and ≤40	≥20 and ≤50
Definition of severe MR	EROA ≥20 mm^2^ and/or RV ≥30 ml	EROA of ≥40 mm^2^ and/or RV ≥60 ml
LVESD		≤70 mm
SPAP		<70 mm Hg
Moderate-severe RV dysfunction	Included	Excluded
**Echocardiographic parameters**		
*MR severity, %*		
Moderate MR (EROA 20–29 mm^2^)	52	14
Moderate-severe MR (EROA 30–39 mm^2^)	32	46
Severe MR (EROA ≥40 mm^2^)	16	41
Mean EROA (mm^2^)	31 ± 10	41 ± 15
Mean LVEDV (ml)	252	192
**Outcomes**		
*Number of clips (%)*		
1 clip	46	36
2 clips	45	55
*Post-procedural moderate-severe MR (%)*		
Post-procedure	9	5
1‑year follow-up	17	5

*HF* heart failure, *LVEF* left ventricular ejection fraction, *MR* mitral regurgitation, *EROA* effective regurgitant orifice area, *RV* regurgitant volume, *LVESD* left ventricular end-systolic dimension, *SPAP* systolic pulmonary artery pressure, *LVEDV* left ventricular end-diastolic volume

First, the sample size of the COAPT trial were larger (614 vs 304 patients). Secondly, there are obvious differences in echocardiographic definition of severe MR, optimal usage of heart failure medication and patient selection between these two studies [23–25]. In the MITRA-FR trial, the mean EROA was 31 mm^2^ and mean RV was 45 ml. More than half (52%) of the patients had an EROA of <30 mm^2^. In contrast, in the COAPT trial, mean EROA was 41 mm^2^. Only 14% of the patients had an EROA of <30 mm^2^. From these findings we can conclude that the definition of severe MR differed between the two studies. The MITRA-FR trial used the 2017 ESC guidelines of valvular heart disease to evaluate MR severity. As we know, severe MR is present when EROA is ≥40 mm^2^ and/or when RV is ≥60 ml. However, for secondary MR it was proposed that these criteria should be less strict due to a substantially higher mortality risk in patients with secondary (ischaemic) MR with an EROA ≥20 mm^2^ or a RV ≥30 ml [26]. In the COAPT trial, severe secondary MR was defined by an EROA ≥40 mm^2^ and/or a RV ≥60 ml. As a result, when applying the original MR grading system, the majority of the patients in the MITRA-FR trial had moderate MR. More specifically, only 16% of the patients in the MITRA-FR trial had an EROA ≥40 mm^2^ vs 41% of the patients in the COAPT trial.

Another difference can be found in the heart failure medication used. In the COAPT trial, heart failure medication was controlled by heart failure specialists from a central committee to ensure that maximum tolerated doses of the medication were taken by the patient before randomisation. Only patients on maximum tolerated doses of heart failure medication were enrolled in the study. When indicated, resynchronisation therapy and/or coronary revascularisation was applied. As a consequence, medication doses were infrequently changed during follow-up. In the MITRA-FR trial, although usage of heart failure medication was guideline-directed, we can assume that not all patients received an optimal dose of heart failure medication. Thus, the use of heart failure medication showed more variability in the MITRA-FR trial, which may more closely represent real-world practice. No details were available about the titration of the medication dose during follow-up.

Also, technical success was higher in the COAPT trial with less residual MR grade ≥3 compared to patients in the MITRA-FR trial (5% vs 9% post-procedure and 5% vs 17% at 12-month follow-up respectively). An important detail is that a second clip was placed more frequently in the COAPT trial (55% vs 45%). Nonetheless, these results should be interpreted with the knowledge that different echocardiographic parameters are used in grading MR and that substantial echocardiographic data are missing at 1‑year follow-up in the MITRA-FR trial.

When looking at additional echocardiographic parameters, it is essential to acknowledge that patients with severe pulmonary hypertension (systolic pulmonary artery pressure >70 mm Hg) and moderate to severe right ventricular dysfunction were excluded from the COAPT trial, but not from the MITRA-FR trial. In addition, a left ventricular end-systolic dimension (LVESD) of >70 mm was an exclusion criterion in the COAPT trial. The mean left ventricular end-diastolic volume (LVEDV) of patients in the MITRA-FR trial was considerably higher than the mean LVEDV of patients in the COAPT trial (252 ml vs 192 ml). The rationale behind the exclusion of patients with a severely dilated LV in the COAPT trial was that these patients were considered to be beyond MV repair. This is based on previous studies which have concluded that severe LV dilatation (left ventricular end-diastolic dimensions of >65 mm and/or LVESD >55 mm) and severe LV dysfunction (LVEF <20%) are associated with less reverse remodelling and a higher mortality rate [27, 28].

## Severe secondary MR: two different entities?

The COAPT trial confirmed that MitraClip implantation is favourable in a specific subset of patients: heart failure with reduced ejection fraction (HFrEF) patients treated with optimal heart failure medication, with severe secondary MR (defined by an EROA of ≥40 mm^2^ and/or a RV ≥60 ml) and a LV which is not severely dilated (LVESD ≤70 mm).

Grayburn et al. proposed a new conceptual framework in response to these trials [29]. It was assumed that secondary MR can be seen as a heterogeneous group with a distinction between ‘proportionate’ MR and ‘disproportionate’ MR. In the MITRA-FR trial, patients had mostly moderate MR (mean EROA 31 mm^2^) with a dilated LV on echocardiography (mean LVEDV of 252 ml). Thus, the severity of MR could be fully explained by the amount of dilatation of the LV. In these patients the severity of MR is proportionate to the degree of LV dilatation. In comparison, patients in the COAPT trial had a mean EROA of 41 mm^2^ with less severe LV dilatation (mean LVEDV 192 ml). As a result, the severity of MR was more than would be expected from the degree of LV dilatation and is therefore called disproportionate MR.

In cases of proportionate MR, the disease primarily involves the left ventricle and it is known that, in these patients, heart failure medication and resynchronisation therapy will lead to better clinical outcomes. Commonly, a reduction of the MR will be seen due to reversal of LV remodelling. Most importantly, a MV intervention will not be more advantageous in this type of patients. LV disease is in this case expected to determine the poor prognosis. On the other hand, in disproportionate MR the primary disease can still be found in the left ventricle. As a result, heart failure medication and resynchronisation therapy remain effective in these patients. Nevertheless, due to the fact that injury of the MV is disproportionate, the severity of MR is more than would be expected. Therefore, it seems logical to believe that percutaneous MV repair will benefit this type of patient.

If it is expected that patients with disproportionate secondary MR will benefit from percutaneous MV repair, how can we differentiate between proportionate and disproportionate MR? In order to answer this question, we first need to realise that the EROA depends on LVEDV and LVEF. In general, patients with HFrEF have a dilated left ventricle. For example, patients with a LVEF of 30% are expected to have a LVEDV of 200–250 ml. In these patients, an EROA of 0.2 cm^2^ is associated with mild MR. Hence, an EROA of ≥0.3 cm^2^ is needed for MR to be classified as severe. Moreover, in patients with more severe LV dilatation (LVEDV >300 ml) an even higher EROA is expected for severe MR. Additionally, EROA is also influenced by the systolic pressure gradient between the left ventricle and the left atrium. In patients with HFrEF and secondary MR, the systolic pressure gradient tends to be low, necessitating an EROA of ≥0.4 cm^2^ in order to reach the criteria for severe MR. As mentioned before, 52% of patients included in the MITRA-FR trial had an EROA <0.3 cm^2^ whereas only 16% presented with an EROA of ≥0.4 cm^2^. Hence, in these patients with proportionate MR more than half had only mild to moderate MR and, as a consequence, a percutaneous MV intervention would not improve the prognosis. Consequently, in patients with a LVEDV of 160–200 ml and an EROA of ≥0.3 cm^2^, the severity of MR is disproportionately high. Most of the patients in the COAPT trial had disproportionate MR with a mean LVEDV of 192 ml and 86% of the patients had an EROA of ≥0.3 cm^2^.

**Fig. 3:**
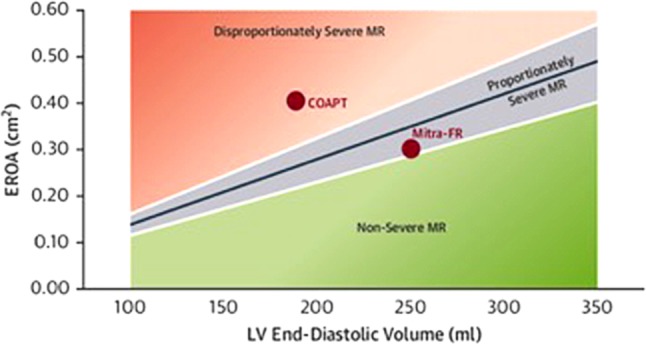
Relationship between effective regurgitant orifice area (*EROA*) and left ventricular end-diastolic volume (*LVEDV*): delineation of ‘disproportionate’ and ‘proportionate’ mitral regurgitation. *LVEF* left ventricular ejection fraction, *RF* regurgitant fraction. Adapted from [29], with permission

In the study of Grayburn et al. [29] an estimation could be made to delineate the relationship between EROA and LVEDV. As a result, proportionate and disproportionate MR can be identified easily (Fig. 3).

## Conclusion

The results of the MITRA-FR and COAPT trials have shown us that percutaneous MV repair can lead to better clinical outcomes in patients with moderate to severe or severe secondary MR. The conflicting results have made us aware that better patient selection is crucial. While optimal dosage of heart failure medication is important, a novel conceptual framework has given us new insights in patients with secondary MR. By dividing patients with secondary MR into those with proportionate MR and those with disproportionate MR, we can better define which patients may benefit from percutaneous MV repair. In the case of proportionate MR, the severity of MR can be linked to the LV dilatation. Therefore, reversal of the LV disease should be targeted as it is expected that the MR itself will not influence the prognosis. In disproportionate MR, the MR severity is worse than would be expected from the LV dilatation. Therefore, the MR should be treated. Primary intervention on the MV itself should have an influence on the prognosis. This hypothesis has been confirmed by the positive outcomes of the COAPT trial, in which primarily patients with disproportionate MR were included in contrast to the neutral outcomes in patients with proportionate MR in the MITRA-FR trial.

Future studies applying this new conceptual framework should give us more clarity as to whether the differentiation between ‘proportionate’ and ‘disproportionate’ MR will indeed lead to consistently better clinical outcomes.
